# Effect of Heat Treatment Processes on the Microstructure and Mechanical Properties of High-Strength Aluminum Alloy Deposited Layers Processed by Fused Arc Additive Manufacturing

**DOI:** 10.3390/ma16206801

**Published:** 2023-10-21

**Authors:** Zhigang Shen, Zhisheng Wu, Ting Wang, Tuosheng Jia, Cuirong Liu

**Affiliations:** 1 School of Materials Science and Engineering, Taiyuan University of Science and Technology, Taiyuan 030024, China; b20190019@stu.tyust.edu.cn (Z.S.); b20190016@stu.tyust.edu.cn (T.J.); 19850019@tyust.edu.cn (C.L.); 2 State Key Laboratory of Advanced Welding and Joining, Harbin Institute of Technology at Weihai, Weihai 264209, China; wangting@hit.edu.cn; 3 Shandong Provincial Key Laboratory of Special Welding Technology, Harbin Institute of Technology at Weihai, Weihai 264209, China

**Keywords:** 7075 aluminum alloy welding wire, TiB_2_ nanoparticle-reinforced phase, T6 heat treatment, tensile strength, corrosion resistance

## Abstract

In this study, 7075 aluminum alloy welding wire with TiB_2_ nanoparticle reinforcement as an additive together with the variable polarity TIG welding arc as a heat source were applied to produce thin-walled deposited layer samples. Results indicated that the performance of the deposited structure of 7075 aluminum alloy with a TiB_2_ reinforcement phase was significantly improved compared to the deposited structure of ordinary 7075 aluminum alloy welding wire. Meanwhile, the precipitation of the TiB_2_ reinforcement phase was insufficient within the structure, and the enhancing effect could not be fully exerted. Moreover, the 7-series aluminum alloy contained a large amount of Zn and Mg elements inside. If the soluble crystalline phase was not fully dissolved, severe stress corrosion could be caused, which inevitably led to a decrease in the mechanical properties. To further improve the performance of the deposited layer, a T6 heat treatment process was performed at 470 °C for 2 h, followed by rapid cooling with distilled water and artificial aging at 120 °C for 24 h. After heat treatment, many second phases appeared in the microstructure of the deposited layer, and the tensile strength increased from (361.8 ± 4.8) MPa to (510.2 ± 5.4) MPa together with the elongation which increased from (9.5 ± 0.5) % to (10.2 ± 0.4) %. The fracture mode of the fracture was a ductile fracture along grain boundaries. The microhardness increased from (145 ± 5) HV to (190 ± 4) HV and exhibited good corrosion resistance in a 3.5% NaCl solution corrosion test.

## 1. Introduction

Wire Arc Additive Manufacturing (WAAM) characterized by high material utilization efficiency, high production efficiency, and low cost is an advanced digital manufacturing technology based on welding technologies such as Melt Inert Gas Shielded Welding (MIG), Tungsten Inert Gas Shielded Welding (TIG), and Plasma Welding (PA) [1,2,3,4]. By automatically adding wires, and under the control of a program, metal parts are gradually formed from a line plane body according to a three-dimensional digital model. Moreover, 7-series aluminum alloys with high specific strength are commonly used in fields such as aerospace and rail transit [5,6,7]. At present, scholars domestically and abroad have achieved relatively mature research results in the application of WAAM technology in the arc additive manufacturing of 5-series and 6-series aluminum alloys. However, research on the arc additive manufacturing of Al-Zn-Mg-Cu (7xxx series) aluminum alloys was still in the exploratory stage. Dong et al. [8] prepared Al-Zn-Mg-Cu alloy using WAAM technology, and a tensile strength of 230 MPa was obtained. The microstructure was composed of columnar grains, with the dispersed distribution of second-phase precipitation. Thomas et al. [9] successfully processed Al-Zn-Mg-Cu alloy using WAAM technology without any hot cracks. In accordance with the research stated above, it could be found that there was a problem of low strength in the deposition layer structure of Al-Zn-Mg-Cu aluminum alloy arc additive manufacturing. Shen et al. [10] used the method of adding TiB_2_ nanoparticle reinforcement to 7075 aluminum alloy welding wire and obtained a crack-free deposition layer structure through the variable polarity TIG fusion wire additive manufacturing process. However, it was found that the tensile strength of the top layer structure of the 7075 aluminum alloy deposition layer with the addition of a TiB_2_ reinforcement phase reached 361.8 MPa and the elongation was (9.5 ± 0.5) %, which was significantly higher than the tensile strength of the conventional 7075 aluminum alloy welding Wire Arc Additive Manufacturing deposition layer (279.4 ± 5.3) MPa [11]. The precipitation of the strengthening phase TiB_2_ inside the structure was insufficient, and the effect of its reinforcement phase was not fully realized. Moreover, the 7-series aluminum alloy contained a large amount of Zn and Mg elements. If the soluble crystalline phase inside the structure was not fully dissolved, the severe stress corrosion could be caused, which inevitably led to a decrease in the mechanical properties. At the same time, corrosion resistance was also a key performance indicator of the 7-series aluminum alloy.

The existing research indicated that the grain size of 7075 aluminum alloy material was increased slightly after heat treatment, and the strength and elongation were improved to a certain extent. The research results of Paul et al. [12] indicated that the Al-Zn-Mg-Cu alloy manufactured using the WAAM process had a tensile strength of up to 400–450 MPa in the deposited layer structure due to the precipitation strengthening after heat treatment.

For 7-series high-strength aluminum alloy materials, the second phase did not have time to precipitate if the heating temperature was above the solid solution line for insulation and rapid cooling, and a supersaturated single α solid solution could be obtained. This process was called quenching. After quenching, the strength and hardness of the aluminum alloy did not immediately change, and the plasticity was better at this point. Due to the relationship between the second phase in the microstructure and α, the degree of coherence of the phases varied. In order to promote the precipitation of the second phase from the supersaturated and unstable α matrix, the second-phase strengthening could be achieved due to the lattice distortion between precipitation and the α. The quenched aluminum alloy needs to be insulated for a period of time under ambient temperature or low-temperature heating conditions. The hardness and strength will be significantly improved with insulation time, while the plasticity will be decreased with insulation time. This process was called aging. However, softening could be caused with respect to the aluminum alloy if the aging temperature was too high or the holding time was too long, and strict control of the aging time and temperature was necessary. The T6 heat treatment process was adopted, which means that the deposited layer sample after wire cutting was firstly subjected to a solid solution strengthening treatment at 470 °C for 2 h. After rapid water cooling, the sample was then subjected to artificial aging treatment at 120 °C for 24 h. The sample after this heat treatment method was called the T6 state sample.

In order to further improve the microstructure and properties of the TiB_2_/7075 aluminum alloy deposition layer, this paper focuses on the influence of the heat treatment process on the microstructure and mechanical properties [13,14,15]. The research results are expected to have important significance for promoting the application of 7075 aluminum alloy arc additive manufacturing technology.

## 2. Materials and Methods

### 2.1. Test Materials

A 7075 B3.5-4S aluminum alloy welding wire with a diameter of 1.2 mm (Shanghai, China) was used as the feeding material. The chemical composition of the welding wire is shown in Table 1 [10]. A 6061 aluminum alloy plate with dimensions of 200 mm × 85 mm × 6 mm (Dongguan, China) was selected as the base plate for the arc additive manufacturing test.

### 2.2. Test Method

#### 2.2.1. WAAM Equipment

**Figure 1:**
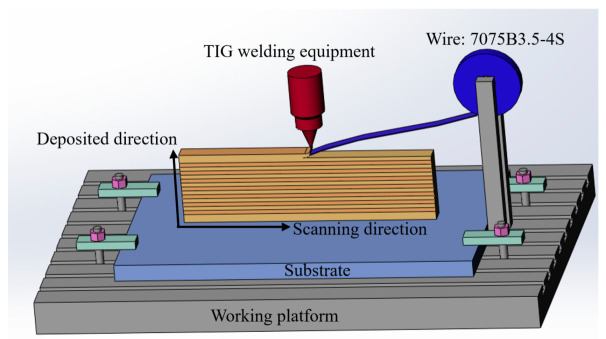
Schematic diagram of the WAAM process.

The WAAM equipment (Weihai, China) is composed of an arc power supply from the welding platform equipped with the variable polarity TIG welding equipment produced by Japan’s Mitsubishi Corporation (Shanghai, China) and the CNC machine tool (Weihai, China). The schematic diagram of the arc additive manufacturing process is shown in Figure 1. The WAAM experimental parameters are listed in Table 2 [10].

#### 2.2.2. Heat Treatment

In order to fully analyze the strengthening effect of the second phase in the sedimentary layer, T6 heat treatment was performed on the sedimentary layer sample, including two stages: solid solution strengthening treatment and aging treatment. Firstly, the samples were heated to 470 °C and held for 2 h, and were then quenched into cold water to an ambient temperature. Finally, the heat preservation was performed at 120 °C for 24 h followed by air cooling.

### 2.3. Analysis and Test Methods

#### 2.3.1. Morphology and Microstructure

The specimens used for metallographic observation were sampled at different positions of the deposited layer of the plate processed by arc additive manufacturing. Then, the microstructure and phase distribution of the samples processed by arc additive manufacturing were observed using a metallographic microscope and scanning electron microscope (Shanghai, China).

#### 2.3.2. Mechanical Test

The surface hardness of the top positions of the as-deposited and the T6 state samples were measured using the Vickers microhardness tester (Shanghai, China).

#### 2.3.3. Grain Morphology

The grain morphology test was conducted using the EBSD (Electron Back-Scattered Diffraction) detection method. The initial processing size of the test sample was 10 mm × 10 mm × 3 mm, and then the test samples were polished using the vibration polishing machine (model: American Buehler Vibromet 2, Lakecraft, IL, USA). The testing equipment consisted of a thermal field emission scanning electron microscope (model: Zeiss GEMINI450, Oberkohen, Germany) and a velocity super EBSD system (model: EDAX, Oxford, UK).

#### 2.3.4. Electrochemical Test

The electrochemical test adopted a three electrode system, with the carbon electrode as the auxiliary electrode, the saturated calomel electrode as the reference electrode, and the prepared sample as the working electrode.

The working electrode size was 15 mm × 15 mm × 3 mm and was connected with copper wire, and the non-working surface was sealed and fixed with epoxy resin. The test surface was polished with water sandpaper of a grade up to 2000. After acetone degreasing and anhydrous ethanol cleaning, it was placed in a dry dish for backup. The test medium was a 3.5% NaCl neutral aqueous solution.

The testing system was composed of the CHI760E electrochemical testing system and related software from Shanghai Chenhua Instrument Co., Ltd. (Shanghai, China). The steady state current potential polarization curve of the test sample was tested with a scanning speed of 0.166 mV/s. After the test, the polarization curve data were analyzed using Corr view software (V2.0). During the EIS test, a sinusoidal disturbance amplitude of 5 mV was applied, and the testing frequency range was 100 kHz~50 mHz. The logarithmic sweep frequency was used to measure a total of 50 points. The Z simp win software (V3.6) developed by the company was used to analyze EIS data and calculate electrochemical equivalent circuit parameters.

## 3. Results and Discussion

### 3.1. Macroforming Characteristics

**Figure 2:**
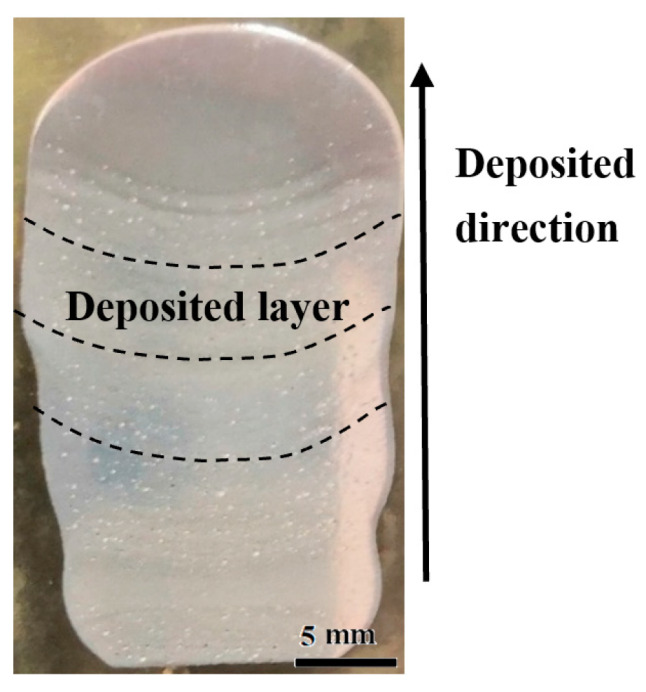
Deposition characteristics of deposited layer.

The welding parameters in Table 2 were used for the manufacturing test of the welding wire arc additive. After polishing the completed deposited layer samples, corrosion was performed and kept for 30 s using Keller reagent. The corroded samples were analyzed by light microscopy. From the macroscopic morphology of the deposited layer in Figure 2, it was observed that there were no crack defects in the 7075 aluminum alloy welding wire arc additive manufacturing deposited layer samples with the TiB_2_ reinforcement phase added. Regarding it as a whole, deposited effect was good.

### 3.2. Microstructure of Sedimentary Layer

**Figure 3:**
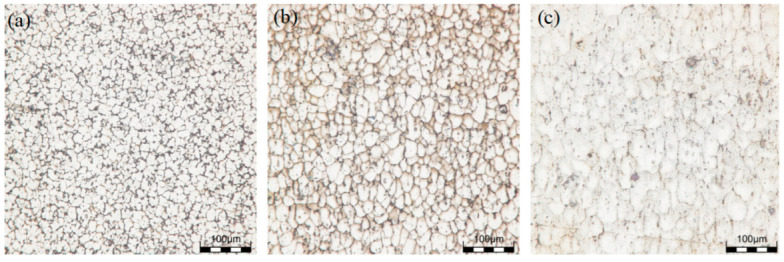
Grain microstructure at different locations of the as-deposited sample: (**a**) the top location; (**b**) the middle location; (**c**) the bottom location.

**Figure 4:**
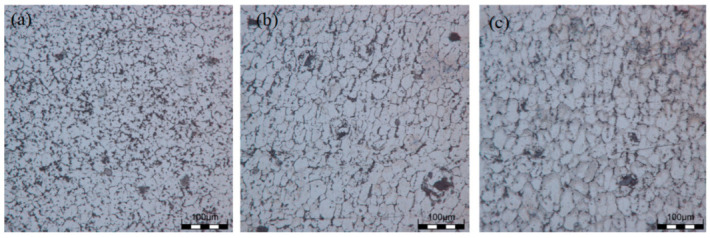
Grain microstructure at different locations of the T6 state sample: (**a**) the top location; (**b**) the middle location; (**c**) the bottom location.

The grain microstructures of the as-deposited samples are shown in Figure 3a–c, and the grain microstructures of the T6 state samples are shown in Figure 4a–c. From the microstructure, it was found that there were more equiaxed grains at the top location, and the second phase was less distributed at the grain boundaries and was mostly clustered at the grain boundaries. After solid solution treatment, α-Al coexisted in a supersaturated state, and the second phase was precipitated from the parent phase after artificial aging. It could be observed that the second phase in the T6 state grain was significantly reduced compared to the deposited grain structure, and precipitation strengthening could be obtained at the grain boundaries due to lattice distortion. The morphology of the middle grain did not undergo significant changes and was similar to the size of the deposited grain. It was evident that a partially continuous second phase appeared at the grain boundary, improving the performance of the specimen. A large number of continuous network second phases were distributed at the bottom grain boundary, and a small amount of second phases were distributed in granular form inside the grains. After EDS analysis, it was found that there was a large distribution of Ti elements in the granular second phase, and a small amount of Mg and Zn elements were distributed. It could be determined that there was a small amount of Mg and Zn elements inside the grains’ η (MgZn_2_) phase, and the second phase was precipitated and accumulates at grain boundaries after heat treatment, which further improved the mechanical properties of the sample.

**Figure 5:**
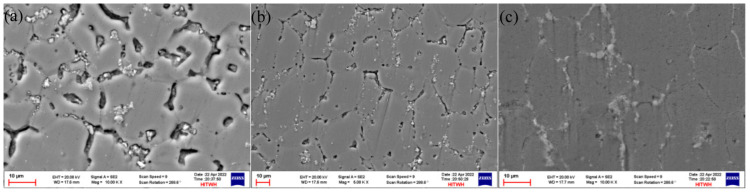
SEM images of the as-deposited sample: (**a**) the top location; (**b**) the middle location; (**c**) the bottom location.

**Figure 6:**
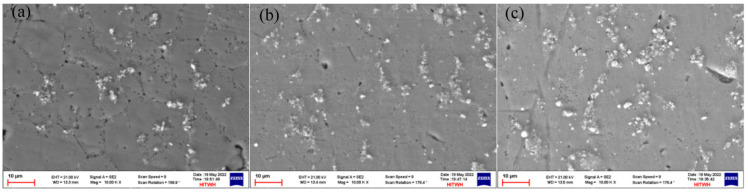
SEM images of the T6 state sample: (**a**) the top location; (**b**) the middle location; (**c**) the bottom location.

**Figure 7:**
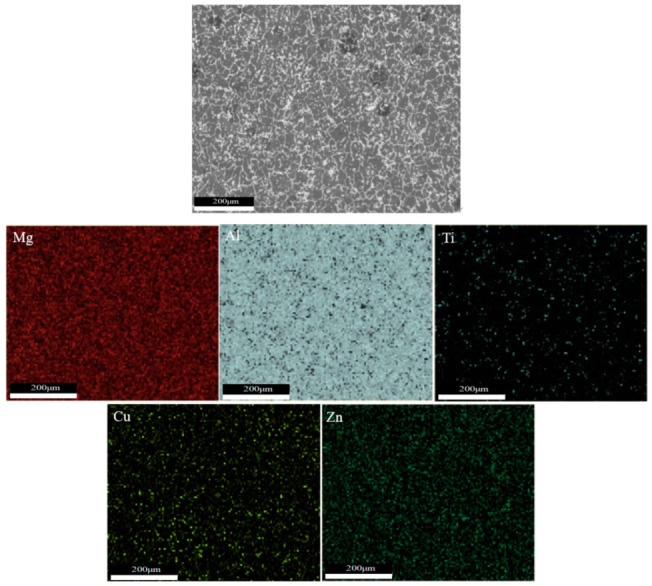
Microstructure and element distribution of as-deposited sample.

**Figure 8:**
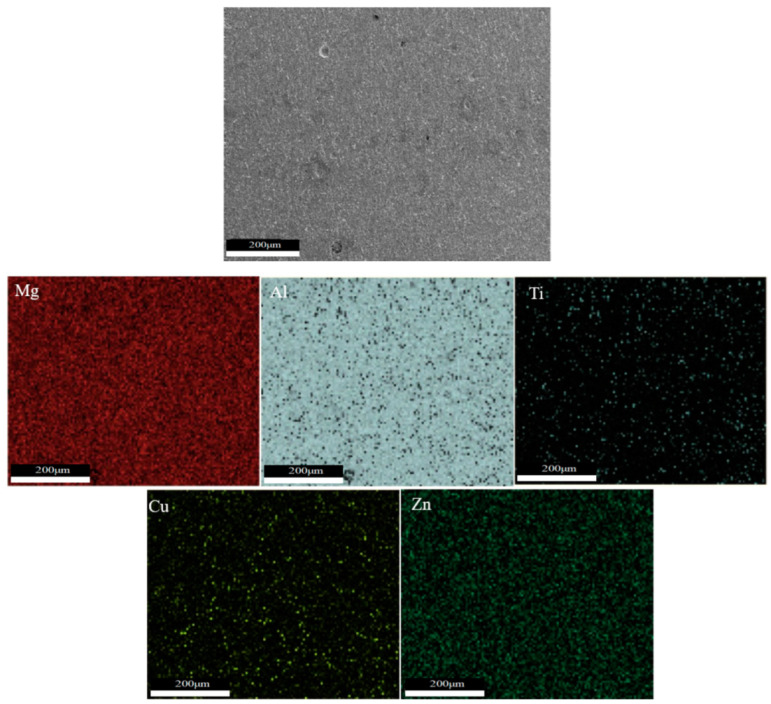
Microstructure and element distribution of T6 sample.

As shown in Figure 5a–c and Figure 6a–c, there are obvious second phases precipitated in the top, middle, and bottom areas of the T6 heat-treated sedimentary layer, and most of them exist in the form of grains. From the elemental scanning results in Figure 7 and Figure 8, it can be seen that Mg, Ti, Cu, and Al elements were enriched, and the density of the Ti element was increased after T6 heat treatment compared to the density before heat treatment, which was related to the precipitation of the second phase to a certain extent.

**Figure 9:**
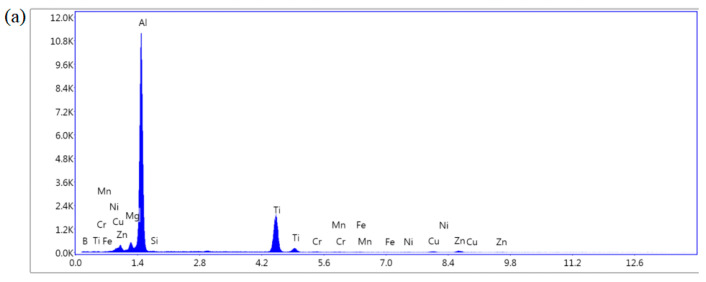

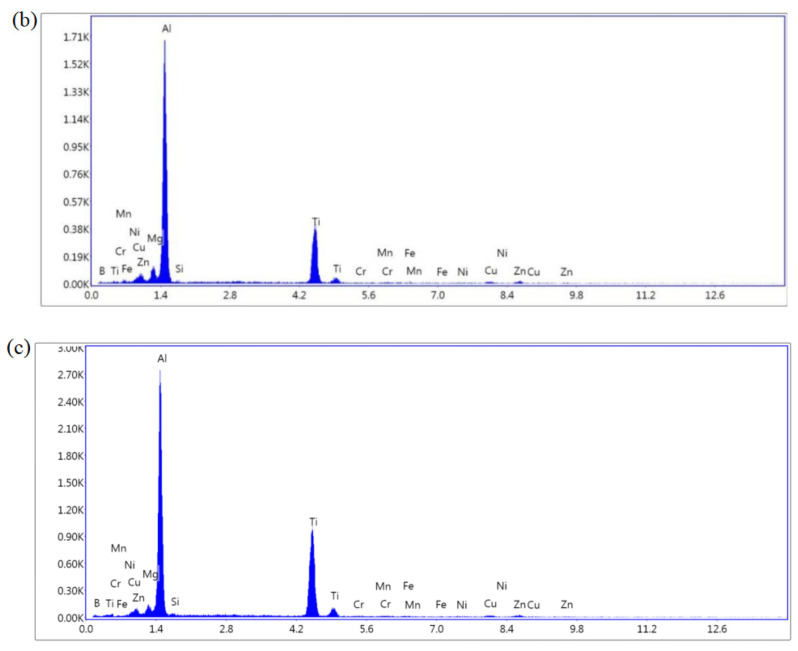
EDS scanning results of the T6 state samples: (**a**) EDS scanning results at the top location of the T6 state sample; (**b**) EDS scanning results at the middle location of the T6 state sample; (**c**) EDS scanning results at the bottom location of the T6 state sample.

As shown in Figure 9, the Ti element content in the bottom structure was the highest. Due to the non-equilibrium solidification inside the molten pool produced by arc additive manufacturing α-Al, the first phase with a high melting point preferentially solidified, while elements such as Ti, Cu, Mg, and Zn inside the structure could aggregate near the solidification interface, which led to element segregation.

After solid solution treatment, these elements dissolved in the form of a eutectic structure and diffused into the Al matrix, which was rapidly cooled by water cooling to form a supersaturated solid solution. The subsequent artificial aging ensured uniform distribution of elements within the microstructure. Moreover, the distribution of these two elements were more uniform due to the faster diffusion rate of Mg and Zn elements inside the aluminum alloy.

### 3.3. Grain Morphology

**Figure 10:**
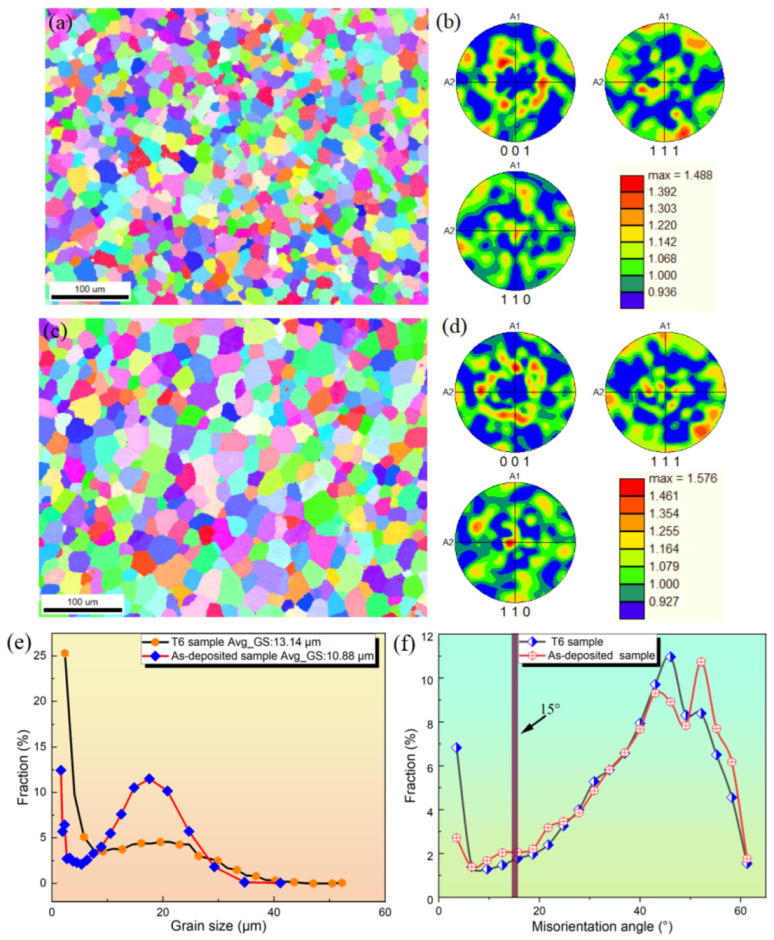
Microstructures of the as-deposited and the T6 state samples: (**a**) IPF map, (**b**,**e**) grain size of the as-deposited sample; (**c**) IPF map, (**d**,**e**) grain size of the T6 state samples; (**f**) misorientation angle of the as-deposited and the T6 state samples.

Grain morphology is one of the factors affecting the properties of aluminum alloys [16]. In order to analyze the microstructures of the deposited layer produced by arc additive manufacturing, the grain morphologies of the deposited and T6 states samples were observed using EBSD. Mechanical grinding was performed on the EBSD samples, followed by electro polishing with a 10% perchloric acid solution and 90% anhydrous ethanol (Shanghai, China) to obtain a smoother and stress-free surface [17]. The EBSD scanning results of the sedimentary layer sample and T6 state sample are shown in Figure 10. The IPF (Image Processing Facility) images of the deposited samples are shown in Figure 10a and the corresponding grain sizes are shown in Figure 10b,e. Figure 10c shows the IPF map of the T6 state samples, and the corresponding grain sizes are shown in Figure 10d,e. Distribution of misorientation angles are shown in Figure 10f.

By observing the IPF images, it was found that the grain morphology of the sample before and after heat treatment was mainly composed of equiaxed grains, with uniform grain size, indicating the structure of the deposited layer was good overall. Generally speaking, obtaining equiaxed grains could be achieved by reducing arc heat input and suppressing grain growth [18]. The main factors affecting grain growth include temperature gradient, solidification rate, and grain orientation. The size and temperature of the melt pool could be reduced by reducing arc heat input, resulting in a low temperature gradient and fast solidification rate, ultimately leading to the formation of equiaxed grains.

### 3.4. Mechanical Tests

#### 3.4.1. Tensile Properties

**Figure 11:**
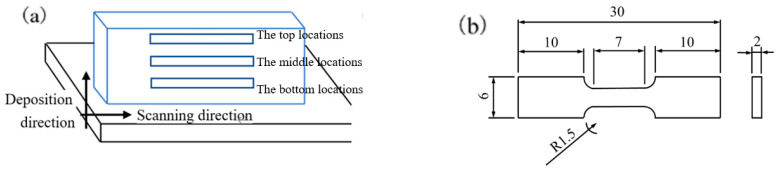
(**a**,**b**) Sampling position and size of tensile samples [10].

Based on the reported research of our team, it was found that the microstructure and properties at the top location of the deposition layer manufactured with a 7075/TiB_2_ aluminum matrix composite welding wire arc additive were the best compared to the middle and bottom regions. Therefore, the microstructure at the top location of the deposition layer was also selected as a representative sample for analysis in this study. Firstly, we took several samples from the top area as shown in Figure 11a, with specific dimensions as shown in Figure 11b [10]. These samples were mainly used for the testing of mechanical properties. At the same time, another sample needed to be taken from the top area of the sedimentary layer to prepare a 10 mm × 10 mm × 3 mm EBSD testing sample. Half of the above two types of test samples were taken for subsequent heat treatment, including two stages of solid solution treatment and artificial aging treatment.

Firstly, the sediment layer samples were subjected to a strengthening treatment of 470 °C for 2 h, followed by rapid water cooling and artificial aging treatment at 120 °C for 24 h. The specimens after this heat treatment process were called T6 state specimens.

After solution treatment and aging heat treatment, the mechanical properties of each specimen had significantly improved. After solid solution treatment, the single α solid solution inside the organization was in a supersaturated state. After aging treatment, the second phase separated out from the α solid solution and grew, and the extrusion of the α phase caused lattice distortion, which had a strengthening effect. After heat treatment, precipitations at the grain boundaries were significantly increased due to the TiB_2_ that was added to the aluminum alloy as the second phase, and the tensile strength of the specimens was improved. From the fracture morphology before and after heat treatment, it could be seen that there were a large amount of uniformly distributed second phases at the grain boundaries after heat treatment. After chemical composition analysis, it was found that there was a large amount of the Ti element involved in the second phase, indicating the strengthening phase could improve the tensile properties of the specimen under the effect of heat treatment.

**Figure 12:**
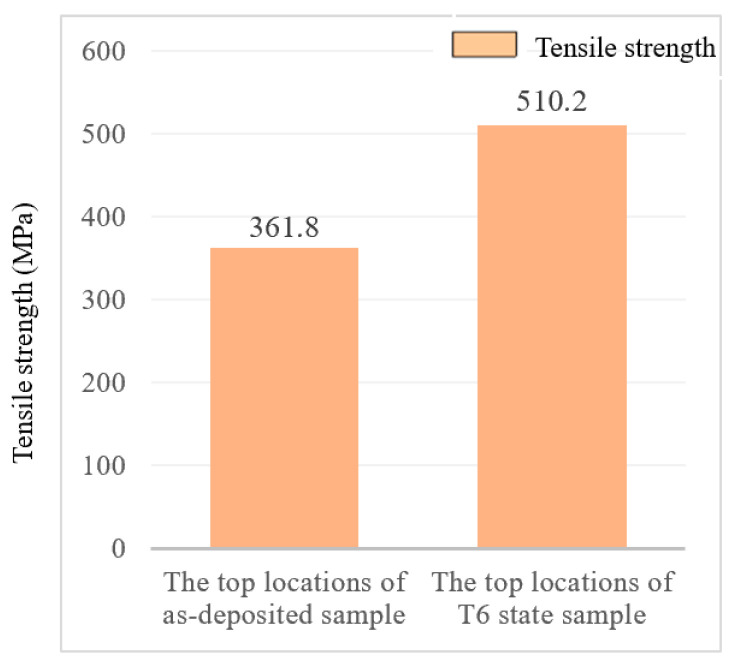
Comparison of tensile strengths at the top locations of as-deposited sample and T6 state samples.

As mentioned above, the top position of the deposited layer was taken for a tensile test, and a tensile strength of (361.8 ± 4.8) MPa and elongation of (9.5 ± 0.5)% were determined. Then, the deposited sample was subjected to T6 heat treatment, resulting in a tensile strength increase of (510.2 ± 5.4) MPa and an elongation increase of (10.2 ± 0.4)% for the T6 state sample. Figure 12 shows the comparison of tensile strengths between the top locations of the as-deposited sample and T6 state samples. From the experimental results, it could be seen that the tensile strength of the deposited sample had been significantly improved after strengthening of heat treatment, with a small increase in fracture elongation. The tensile strength had been increased by 41.02%, and the elongation had only been increased by 7.37%.

**Figure 13:**
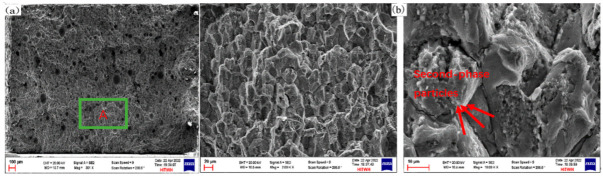

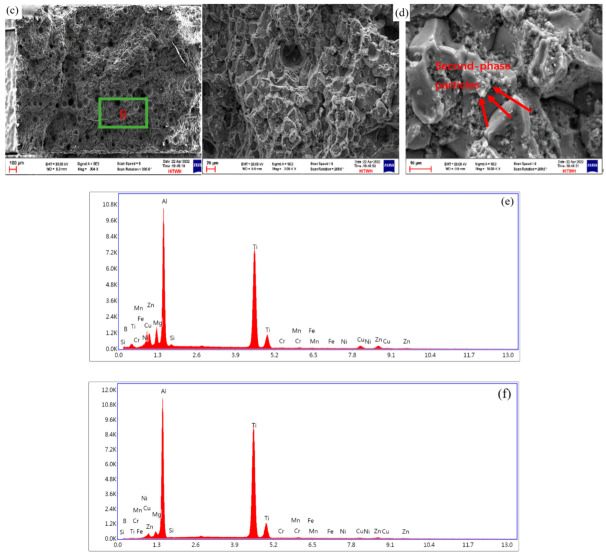
EDS scanning result of fracture morphology of as-deposited and T6 state tensile samples: (**a**) fracture morphology of as-deposited sample; (**b**) local amplification of zone A [10]; (**c**) fracture morphology of T6 state sample; (**d**) local amplification of zone B; (**e**) EDS scanning result of fracture morphology of as-deposited tensile sample; (**f**) EDS scanning result of fracture morphology of T6 tensile sample.

Figure 13 shows the comparison of tensile fracture morphology between the deposited (Figure 13a,b) [10] and heat-treated T6 state samples (Figure 13c,d). Observing the fracture morphology of the T6 state samples, it was found that the appearance of polycrystalline rocks and rock sugar patterns could be seen. The interface mostly showed a smooth morphology, and the fracture surface presented a particle shape, indicating the occurrence of a brittle fracture along the grain boundaries. Compared to the deposited sample, the second phase separated out from the α solid solution and grew, and the extrusion of the α phase caused lattice distortion after T6 heat treatment. Similarly, the precipitation of the second phase at the grain boundary of the T6 state sample was significantly increased compared to the deposited fracture surface, which further improved the tensile properties of the T6 state sample and allowed the second phase in the grain to fully play its role.

**Figure 14:**
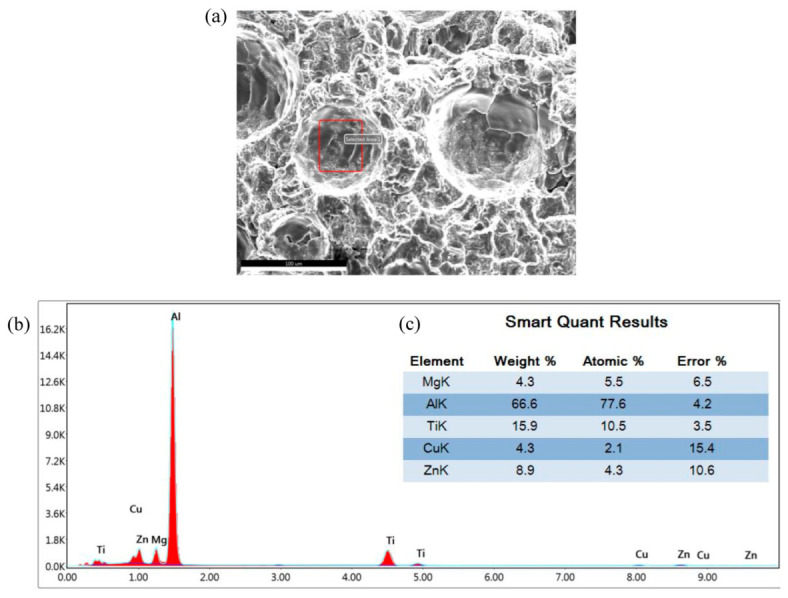
The morphology of tensile fracture dimples and EDS scanning results: (**a**) dimple morphology; (**b**,**c**) EDS scanning results.

The uniform distribution of dimples on the fracture surface of the tensile sample is the most typical feature of T6 state samples. As shown in Figure 14a, there are many dimples distributed on the tensile fracture morphology of the T6 state sample, and there are residual nano second-phase particles at the bottom of the dimples. As shown in Figure 14b,c, EDS scanning analysis of the dimples revealed the presence of a high content of the Ti element, suggesting that TiB_2_ nanoparticles played a strengthening role. Research has shown that during the stretching process, the second-phase particles are harder, leading to the first deformation of the softer Al matrix, forming micro voids. After fracture, the characteristic morphology of the dimples appears as a transgranular fracture mode [19,20]. Therefore, the fracture mode of the T6 state sample is a ductile fracture, which is consistent with the conclusion that the elongation after fractures slightly increases after heat treatment.

#### 3.4.2. Microhardness

**Figure 15:**
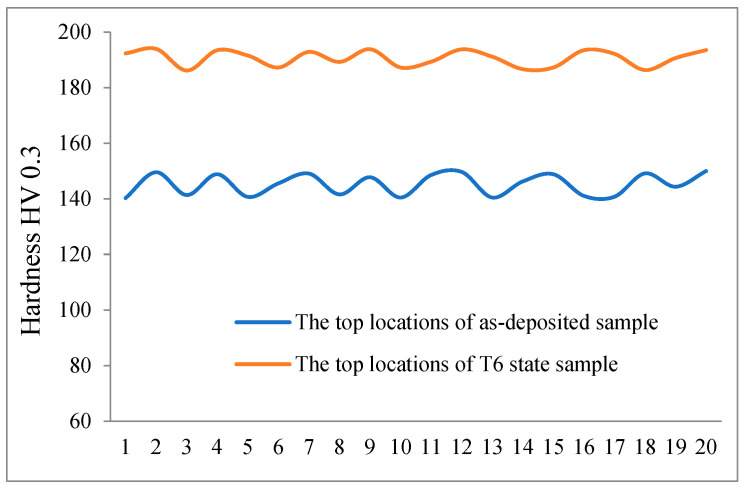
Microhardness curves of the as-deposited and the T6 state samples.

After T6 heat treatment, the second phase in the deposited layer undergoes supersaturated precipitation, and the internal elements were more evenly distributed under aging treatment. The microstructure was strengthened, which could result in a significant increase in the microhardness of the T6 state sample. Microhardness curves of the as-deposited and the T6 state samples are shown in Figure 15, and it was found that the microhardness at the top location of the deposited layer sample increased from (145 ± 5) HV to (190 ± 4) HV, indicating a good heat treatment effect.

#### 3.4.3. Corrosion Resistance

Ti elements could strengthen 7075 aluminum alloy and significantly improve its stress corrosion resistance, which is closely related to its ability to significantly improve the microstructure of the aluminum alloy. The first was the role of grain refinement. Ti was one of the commonly used grain refiners in aluminum alloys. Adding elements such as Ti and B to the aluminum alloy melt would form a large number of Al_3_Ti and TiB_2_ dispersed particles, and their lattice constants related to α (Al) were close and had a good coherent relationship, which could act as the core of heterogeneous nucleation in aluminum alloys. In this research, the 7075 aluminum alloy welding wire used had already added TiB_2_ nanoparticle reinforcement before the arc additive manufacturing, which also had the same strengthening effect.

After T6 treatment, the strength and hardness of 7075 alloy containing TiB_2_ reached their maximum values. Therefore, it was necessary to confirm its electrochemical corrosion resistance. In order to investigate the effect of heat treatment on the corrosion resistance of TiB_2_/6056 aluminum matrix composite additive parts, Feng [21] conducted electrochemical polarization and electrochemical impedance corrosion studies on the additive samples in the deposited and heat-treated states, respectively. The results showed that the corrosion resistance of the additive was significantly improved in a 3.5wt% NaCl solution after heat treatment.

Therefore, we did not conduct comparative tests on the corrosion resistance of TiB_2_/7075 aluminum matrix composite samples in the deposited state and T6 state, but instead chose three heat-treated samples at different solid solution temperatures for comparison of corrosion resistance. In order to compare the effect of heat treatment solution temperature on its corrosion performance, we conducted solution treatments at three different temperatures, including 460 °C (2 h), 470 °C (2 h), as well as 480 °C (2 h). The temperature and time of aging treatment were consistent with T6 heat treatment (120 °C, 24 h).

**Figure 16:**
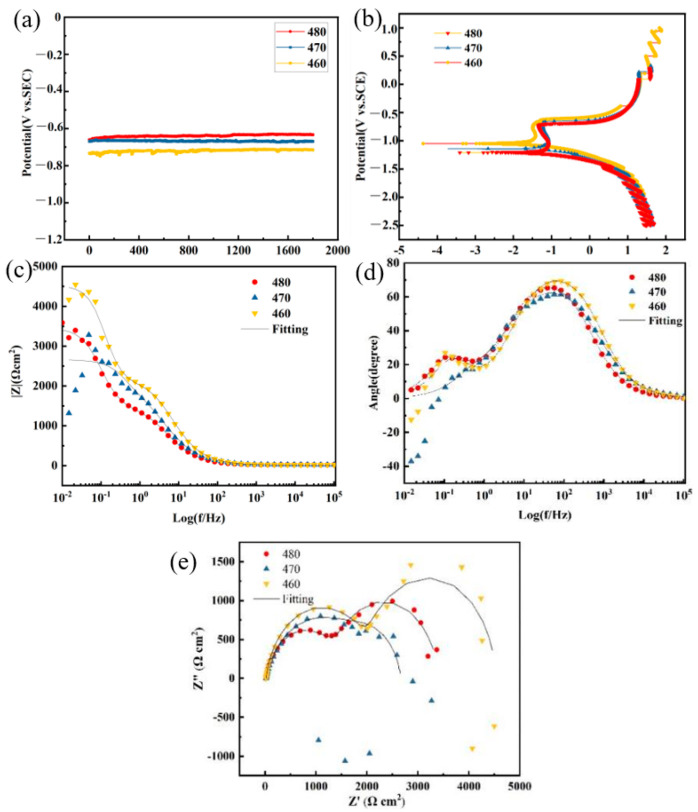
Open circuit, polarization, and impedance diagrams of electrochemical tests: (**a**) open circuit comparison diagram; (**b**) polarization comparison diagram; (**c**) bode diagram; (**d**) phase angle comparison diagram; (**e**) impedance comparison diagram.

Corrosion resistance was a key performance indicator of the 7-series high-strength aluminum alloy material. The test sample was immersed in a test solution (3.5% NaCl solution) (corrosive environment) for 24 h. Comparative data on the corrosion resistance of deposited specimens under different heat treatment temperatures were obtained, shown in Figure 16.

The open circuit test of electrochemical testing was conducted without current flow. When observing the potential value of the test sample, the larger the potential value, the greater the initial surface corrosion tendency of the test sample, which indicates that the corrosion resistance of the sample was the worst. From the curve in Figure 16a, it could be inferred that the corrosion resistance of the 460 °C heat-treated sample was better compared to the 470 °C and 480 °C heat-treated samples.

From the polarization comparison diagram in Figure 16b, it could be seen that the potential value of the 460 °C heat-treated sample was more to the left and higher compared to the 470 °C and 480 °C heat-treated samples. This also indicated that the corrosion resistance of the 460 °C heat-treated sample was better compared to the 470 °C and 480 °C heat-treated samples. The potential was further to the left and the current density was smaller, so it could be inferred that the corrosion resistance of the 460 °C heat-treated sample was the best.

The larger the impedance value Z, the better the corrosion resistance of the test sample. From the bode diagram in Figure 16c, it could be seen that the impedance value of the 460 °C heat-treated sample was higher compared to the 470 °C and 480 °C heat-treated samples, which also indicated that the corrosion resistance of the 460 °C heat-treated sample was better than that of the 470 °C and 480 °C heat-treated samples.

The peaks in the phase angle in Figure 16d correspond to the capacitance arc in Figure 16e, with the maximum phase angle being the 460 °C heat-treated sample and the minimum phase angle being the 470 °C heat-treated sample. A smaller phase angle indicated poorer corrosion resistance. Therefore, it could be inferred that the corrosion resistance of the 460 °C heat-treated sample was the best.

The magnitude of the impedance value Z in Figure 16e represented the corrosion rate of the deposited layer sample. The larger the capacitance arc radius, the better the corrosion resistance. Therefore, it could be inferred that 460 °C had the best corrosion resistance.

From the above corrosion resistance test results, it can be seen that the corrosion resistance of the deposited layer sample treated at 460 °C was better compared to the 470 °C and 480 °C, which meant that the corrosion resistance was decreased with the increase in heat treatment melting temperature.

## 4. Conclusions

This article mainly studies the effects of the T6 heat treatment process on the microstructure, mechanical properties, and corrosion resistance of the 7075 aluminum alloy arc additive deposition layer with a TiB_2_ reinforcement phase added. Based on the experimental results and analysis, the following conclusions were obtained:

(1) After T6 heat treatment (470 °C, 2 h) with solid solution strengthening treatment and artificial aging treatment (120 °C, 24 h), the mechanical properties of the T6 state specimen were significantly improved.

(2) After heat treatment, the eutectic structure at the grain boundaries were dissolved and disappeared, which entered the interior of the grains in the form of particles. The distribution of the second phase inside the microstructure was uniform. The distribution of alloy elements was uniform, and the phenomenon of element segregation was reduced.

(3) The tensile performance of the T6 state specimen was significantly improved, the tensile strength increased from (361.8 ± 4.8) MPa to (510.2 ± 5.4) MPa, the microhardness increased from (145 ± 5) HV to (190 ± 4) HV together with the elongation which increased from (9.5 ± 0.5)% to (10.2± 0.4)%. The fracture mode of the T6 state specimen was a ductile fracture, which is consistent with the conclusion that the elongation after fracture slightly increases after heat treatment.

(4) In the deposited state, there were TiB_2_ particles and second-phase compounds in the aluminum matrix composite material, and TiB_2_ particles and compound phases were clustered at the intergranular positions, which significantly damages the continuity of the Al_2_O_3_ oxide film on the material surface, which also provides a possibility for corrosion. At the same time, the refinement of the compound phase after heat treatment also significantly improves the continuity of the Al_2_O_3_ oxide film on the surface of the additive, which provides better protection for the additive sample. These reasons lead to a significant improvement in the corrosion resistance of the additive after heat treatment.

## Figures and Tables

**Table 1:** Chemical composition of 7075 aluminum alloy welding wire (%, mass fraction) [10].

	Zn/wt.%	TiB_2_/wt.%	Mg/wt.%	Ti/wt.%	Cu/wt.%	Mn/wt.%	Cr/wt.%	Si/wt.%	Al/wt.%
Welding wire	5.52	3.5–4	2.3	0.1	1.4	0.3	0.22	0.2	Bal.

**Table 2:** Arc additive manufacturing process parameters [10].

Sample	Current (A)	Traveling Speed (mm/min)	Wire Feeding Speed (cm/min)	Current Model	Quantity of Position Layer
1#	180	220	100	AC square wave	40

## Data Availability

Not applicable.
